# Peer victimization and experiences of violence at school and at home among school age children with disabilities in Pakistan and Afghanistan

**DOI:** 10.1080/16549716.2020.1857084

**Published:** 2020-12-24

**Authors:** Rozina Somani, Julienne Corboz, Rozina Karmaliani, Esnat D. Chirwa, Judith McFarlane, Hussain Maqbool Ahmed Khuwaja, Nargis Asad, Yasmeen Somani, Ingrid Van Der Heijden, Rachel Jewkes

**Affiliations:** a Faculty of Nursing, University of Toronto, Toronto, Canada; b Gender and Health Research Unit, South African Medical Research Council, Cape Town, South Africa; c School of Nursing & Midwifery and Community Health Sciences, Aga Khan University, Karachi, Pakistan; d School of Public Health, University of Witwatersrand, Johannesburg, South Africa; e College of Nursing, Texas Woman’s University, USA; f School of Nursing & Midwifery, Aga Khan University, Karachi, Pakistan; g Department of Psychiatry, Aga Khan University, Karachi, Pakistan

**Keywords:** Disability, violence against children, peer violence victimization, peer violence perpetration

## Abstract

**Background**: Children with disabilities are more likely to experience violence or injury at school and at home, but there is little evidence from Central Asia.

**Objective**: To describe the prevalence of disability and associations with peer violence perpetration and victimization, depression, corporal punishment, school performance and school attendance, among middle school children in Pakistan and Afghanistan.

**Method**: This is a secondary analysis of data gathered in the course of evaluations of interventions to prevent peer violence conducted in Pakistan and Afghanistan as part of the ‘What Works to Prevent Violence against Women and Girls Global Programme’. In Pakistan, the research was conducted in 40 schools, and disability was assessed at midline in 1516 interviews with Grade 7s. In Afghanistan, the data were from the baseline study conducted in 11 schools with 770 children. Generalized Linear Mixed Modeling was used to assess associations with disability.

**Results**: In Afghanistan, the prevalence of disability was much higher for girls (22.1%) than boys (12.9%), while in Pakistan 6.0% of boys and girls reported a disability. Peer violence victimization was strongly associated with disability in Afghanistan and marginally associated in Pakistan. In Pakistan, perpetration of peer violence was associated with disability. In both countries, disability was significantly associated with higher depression scores. Food insecurity was strongly associated with disability in Afghanistan.

**Conclusion**: Disability is highly prevalent in Afghanistan and Pakistan schools and this is associated with a greater risk of experiencing and perpetrating peer violence. It is important to ensure that all children can benefit from school-based prevention interventions.

## Background

The United Nations Convention on the Rights of Person with Disabilities defines disabilities in children as any kind of physical, sensory, intellectual, mental, or functional impairment which may cause barriers to participation in academic and/or social activities [1]. The World Health Organization (WHO) estimates that 15% of the world’s population live with some form of disability [2], including 93 million children. Yet, due to varying definitions, and limited use of comprehensive, validated measurement tools, estimates of the prevalence of disabilities in children in low- and middle-income countries (LMICs) vary widely [3]. Although the World Report on Disability estimates that 5 million people live with disabilities in Pakistan [2], there are no reliable estimates of disability among children in Pakistan [4], although research suggests that the prevalence of intellectual disabilities among children in Pakistan may be higher than the global average [5].

The National Disability Survey Afghanistan, conducted in 2005, suggested that the prevalence of disability was between 2.7% and 4.7%, depending on definition [6]. However, the 2007/8 National Risk and Vulnerability Assessment suggested that the prevalence of disability in Afghanistan was lower at 1.6% [7], but that the largest number of disabled persons (57,000) were aged 10–19 years [7]. In contrast, the International Committee of the Red Cross has estimated that 1 million children in Afghanistan have been disabled due to war and conflict [8]. Recent estimates for all disability levels among children aged 2–17 years in Afghanistan are as high as 17.3% [9].

The relationship between disability and violence is reciprocal: while disability increases the risk of violence exposure, experiencing acts of violence can lead to disability [10]. Children with disabilities are structurally disempowered, stigmatized and socially disadvantaged, and four times more likely to experience violence than non-disabled children [11]. Cognitively impaired children are at higher risk of sexual abuse, with their vulnerability compounded by a lack of basic information and education about sexual health [11]. Girls with disabilities are at increased risk of violence because of their gender and disability [12]. Disabled students are significantly more likely to experience violence or injury from school staff and physical or emotional violence or bullying by peers [13]. Childhood experience of violence may lead to high-risk behaviors, physical and psychosocial health problems, and exacerbate preexisting disabilities [14–16].

Children with disabilities are also more vulnerable to mental ill-health. Children with learning disabilities have poorer emotional wellbeing and a greater risk of loneliness, depression, anxiety, feelings of rejection and low self-esteem [17–19]. Understanding the experiences of disabled children in school settings is important, especially in LMICs such as Pakistan and Afghanistan where data to inform public policy and prevention interventions are lacking. In order to better understand the prevalence of violence against children with disabilities, we conducted a secondary analysis of data from two studies conducted with school children in Pakistan and Afghanistan. These studies are part of a larger global initiative that aims to discover what works to prevent violence against women and girls [20].

The objectives of the paper are to estimate disability prevalence among students in secondary schools and to describe associations between having a disability and peer violence perpetration and victimization, corporal punishment, depression, school performance and school attendance. To our knowledge, this is the first comparative study of its type on the prevalence of disability among school students and the relationship between disability and violence in school-based settings in Pakistan and Afghanistan.

## Methods

In Pakistan and Afghanistan, data were collected as part of two separate, but comparable, studies of violence prevention interventions conducted with school children. This paper reports a secondary analysis of these studies. These studies are reported in detail elsewhere [21–25]. In Pakistan, baseline data were collected from 6^th^ grade children (930 girls and 822 boys) in 20 girls’ and 20 boys’ schools in Hyderabad City, Sindh Province who were participating in an intervention evaluation to assess the effectiveness of an intervention to prevent peer violence. All students agreed to participate and had parental consent. In Afghanistan, baseline data were collected from 7^th^ and 8^th^ grade students (420 girls and 350 boys) in 11 schools (seven girls’ schools and four boys’ schools) in three districts of Jawzjan province who were participating in an intervention study to measure the effectiveness of a school and community-based peace education programme in reducing violence.

**Figure 1.**
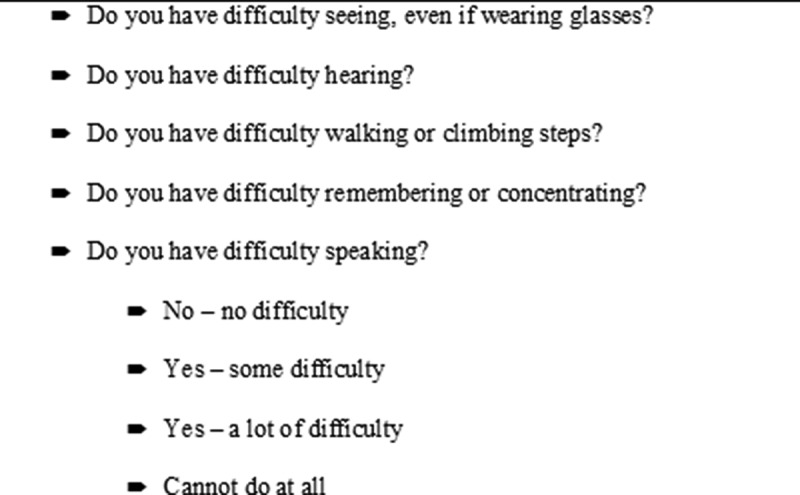
The Washington group on disability statistics questionnaire

Similar questionnaires were used in both countries. To assess disability, the study used the modified Washington Group on Disability Statistics questionnaire (Figure 1), comprising five domains: vision, hearing, walking/movement, memory/concentration, and speech. Difficulty was measured as ‘no difficulty’, ‘some difficulty’, ‘a lot of difficulty’, and ‘cannot do at all’. Students were identified as disabled if they reported ‘a lot of difficulty’ or ‘cannot do at all’ in any domain. However, the study in Pakistan was commenced a little before the study in Afghanistan, with the result that the disability questions were not included at baseline in Pakistan, but were rather added for the midline assessment, 12 months after baseline. For this analysis we assume disability reports to be unchanged and have merged the variable on disability into the baseline dataset from Pakistan. In Afghanistan, all assessments were at the same time point (baseline).

Peer violence victimization was measured using the Peer Victimization Scale [26], which consists of 16 items, covering self-reported physical victimization, verbal victimization, social manipulation, and property attacks in the prior month. Students were coded as having experienced peer victimization if they reported more than one type or episode of violence in the past month, in line with the Center for Disease Control (CDC) definition of peer violence [27]. A Peer Perpetration Scale was developed for both studies by adjusting the wording of the Peer Victimization Scale to measure peer perpetration. The coding strategy outlined above was applied.

Students’ depression was measured with the Children’s Depression Inventory (CDI-2), which asked them to rate their feelings in the past 2 weeks in response to 28 items [28]. Each item has three possible responses: (1) ‘I have fun in many things’, (2) ‘I have fun in some things’, or (3) ‘Nothing is fun at all’. Each item was given a value from 0 to 2, and scale scores were computed by summing item responses. Aggregated scores ranged between zero and 56, where higher values indicate greater severity of depressive symptoms.

Corporal punishment at school in the last month was measured using six items and physical punishment at home in the last month using two items. We also assessed the students’ exposure to family violence in the past month with one item on witnessing their mother being beaten by their father, one on her being beaten by other relatives, and one on their father fighting with another man. All these variables were included in the analysis binary measures (ever in the last 4 weeks v. never).

School performance was measured in different ways across the two studies. In Pakistan, it was assessed through four questions covering performance in mathematics, science, language, and Pakistan studies (below average, average, and above average). In Afghanistan, students were administered simple literacy and numeracy tests, including reading one line of the questionnaire, and completing three simple sums. For literacy, students were coded on a scale of one to four, i.e. not able to read at all, reading with difficulty, reading with little difficulty, or reading fluently. For numeracy, students were coded on a scale of one to four, i.e. not numerate at all, adding with difficulty, adding with ease or dividing with ease. Literacy and numeracy items were summed to give an overall school performance score.

## Data analysis

The Pakistan data were prepared by merging the disability measures from midline into the baseline dataset. Missing data were assessed for items of every measure during the data collection process. In Pakistan, the overall percentage of missing data ranged from 0.2% to 0.7%. If a student did not respond to all items on peer violence perpetration or victimization, an additive score was derived from items that the student responded to and this was then dichotomized. In Afghanistan, only one case had some missing data in two measures. No imputation of missing data was done for either study. We examined the data by gender but decided to pool the analysis as the findings for boys and girls were very similar, but power was limited in Pakistan for a gender-disaggregated analysis due to the low prevalence of disability.

Frequencies and percentages were used to summarize binary or categorical variables, while means and standard deviations were used to summarize continuous variables. Generalized linear mixed effects modeling was used to assess the relationship between disability (as an exposure) and depression, peer victimization, peer perpetration, experiencing corporal punishment at school and school performance/attendance. We fitted three models for each outcome to assess the relationship between disability and other outcomes that were measured in the study. The first model had disability as the only exposure and the second had disability as the exposure while adjusting for students’ age and sex; the third model included other explanatory variables previously associated with the outcomes. For peer violence perpetration or victimization models, the exposures included hunger/food insecurity, witnessing violence against mother perpetrated by father or other relatives, experiencing physical punishment at home, and school performance [24]. The model for depression had similar exposures as the model for peer violence perpetration or victimization, but also included students’ experience or perpetration of violence as an exposure [29]. All analyses took into account the designs of the two samples, with the students clustered within schools. Statistical significance was set at p < 0.05. All analyses were undertaken using Stata version 14.

## Results

Most students were girls (Table 1) and about 60% of participants in Pakistan were 12 years or younger compared to only 6% in Afghanistan. Most students in Afghanistan were 14 years or older (52.6%) compared to only one-fifth (18.1%) of students in Pakistan. Food insecurity was common and not very different between the two countries, and most of the children lived in large families.

**Table 1.** Socio-demographic characteristics of study participants

	Pakistan (N = 1516)		Afghanistan (N = 770)	
	Mean/n	sd/%	Mean/n	sd/%
Gender (% girls)	835	55.1	420	54.5
Age group of learner (%)				
≤12 yrs	928	61.2	47	6.1
13 yrs	314	20.7	149	19.4
14 yrs	150	9.9	256	33.2
≥15 yrs	124	8.2	318	41.3
Food secure (%)	1062	70.1	640	83.1
Family size				
Average number in HH	9.8	5.6	9.2	3.0
Average number of brothers	2.4	1.5	3.4	1.6
Average number of sisters	2.4	1.7	3.3	1.7

The proportion of students with disability questions is shown in Table 2. In Pakistan, 91/1516 participants (6%) reported any kind of disability, compared to 138/770 (17.9%) in Afghanistan. Some participants reporting more than one type of disability; thus, 174 disabilities were recorded across the 138 Afghan students with disabilities. The prevalence of disability in Afghanistan was much higher for girls (22.1%) than boys (12.9%), but this difference was not seen in Pakistan (6.0% in boys and in girls). In both countries, memory/concentration problems were the most common type of disability. The second most frequently reported disability in Pakistan is related to movement and, in Afghanistan, to vision.

**Table 2.** Prevalence of disability in the samples of students from Pakistan and Afghanistan

Type of disability	Pakistan (n = 1516)	Afghanistan (n = 770)
Speech	10 (0.7)	26 (3.4)
Vision	16 (1.1)	37 (4.8)
Hearing	10 (0.7)	19 (2.5)
Movement	24 (1.6)	13 (1.7)
Memory/concentration	53 (3.5)	79 (10.3)
Any disability	91 (6.0)	138 (17.9)

In both countries, students with a disability had lower school performance scores and on average, missed more days of school (Table 3). Levels of peer violence (perpetration or victimization) were generally higher in Pakistan than in Afghanistan. However, in both countries, students with disabilities had higher peer violence victimization scores, and in Pakistan, they had higher peer violence perpetration scores. Furthermore, in both countries, substantial co-occurrence of perpetration and victimisation were found, such that a large proportion of students (particularly disabled students) who perpetrated peer violence were also victimized. Depression levels were generally higher in Afghanistan than in Pakistan, and students with disabilities had higher depression levels. In Afghanistan, almost half of the students (47.1%) with a disability experienced corporal punishment at school, compared with almost 40% of non-disabled children. A higher proportion of students experienced corporal punishment at school in Pakistan compared with Afghanistan (59.0% vs 39.4%) and the proportion of disabled students in each setting experiencing it was higher than non-disabled. The proportion of students experiencing physical punishment at home in Pakistan was higher than in Afghanistan (47.0% vs 18.4%). However, in both countries it differed little by disability.

**Table 3.** Descriptive statistics of school characteristics (school performance and attendance), depression, violence experience or witnessing, disaggregated by disability status

	Pakistan		Afghanistan	
	No disability (n = 1425)	Disability (n = 91)	No disability (n = 632)	Disability (n = 138)
Outcomes/factors	n or mean (%/sd)	n or mean (%/sd)	n or mean (%/sd)	n or mean (%/sd)
**School factors**				
School performance score	9.5 (1.8)	8.9 (1.7)	4.9 (1.1)	4.1 (1.4)
Mean days school absence in last month	3.5 (3.5)	4.0 (4.1)	2.3 (2.2)	2.6 (2.6)
**Mental health**				
Depression score	9.7(5.7)	13.0(7.4)	12.9 (6.8)	14.1 (5.8)
**Experiencing/witnessing violence**				
Peer violence perpetration score	4.9(6.3)	7.9(8.9)	1.16 (2.7)	1.26 (2.7)
Peer violence victimization score	9.4(8.5)	12.0(8.6)	2.85 (4.6)	5.01 (6.3)
Both peer violence victimization and perpetration. **§**	698 (48)	57 (62.6)	312 (49.4)	87 (63)
Experienced corporal punishment at school. **§**	836 (58.7)	58 (63.7)	238 (37.7)	65 (47.1)
Experienced physical punishment at home. **§**	671 (47.1)	41 (45.1)	111(17.6)	31 (22.5)
Witnessed father fight with other men. **§**	298 (20.9)	29 (31.9)	62 (9.8)	17 (12.3)
Witnessed mother being beaten by father. **§**	101(7.1)	11(12.1)	19 (3)	8 (5.8)
Witnessed mother being beaten by other family member. **§**	58(4.1)	4(4.4)	12(1.9)	5 (3.6)
Household food security score (high = more insecure)	0.5(0.9)	1.1(1.4)	0.3(0.8)	0.6(1.5)

**§**Summary statistics represented by frequencies and percentages.

In both Pakistan and Afghanistan, a higher proportion of students reporting having witnessed their father fight with other men was higher among those with a disability (31.9%) than those without (31.9% v. 20.9% in Pakistan, and 12.3% v. 9.8% in Afghanistan). Similarly, in both countries, a higher proportion of disabled than non-disabled students had witnessed their mothers being beaten by their father. In both Pakistan and Afghanistan, children with disabilities were much more food insecure than those without. This was particularly marked in Pakistan.

Generalized linear regression models showing factors associated with students having a disability are presented in Table 4. In both countries, disability was associated with significantly higher depression scores. The estimated mean difference in depression scores between disabled and non-disabled students decreased slightly when adjusting for potentially confounding factors; however, the difference in depression scores between the two groups remained highly significant (Pakistan: EMD = 2.3, p-value<0.001; Afghanistan: EMD = 1.5, p-value = 0.003). There was no association in both countries between disability and experience of corporal punishment at school.

**Table 4.** General linear regression models: different outcomes with disability as one of the exposures

		Pakistan				Afghanistan			
Outcomes	Model	EMD/OR	LCL	UCL	p-Value	EMD/OR	LCL	UCL	p-value
Depression score	1	3.8	2.6	5.0	<0.001	2.1	1.1	3.1	<0.001
	2	3.8	2.6	5.0	<0.001	2.2	1.2	3.1	<0.001
	3	2.3	1.2	3.4	<0.001	1.5	0.5	2.4	0.003
Corporal punishment at school§	1	1.2	0.7	2.2	0.487	1.5	0.9	2.5	0.149
	2	1.3	0.7	2.5	0.403	1.6	1.0	2.5	0.042
	3	0.8	0.5	1.4	0.461	1.3	0.7	2.4	0.481
Peer violence victimization score	1	2.6	0.9	4.3	0.003	2.1	1.3	3.0	<0.001
	2	2.6	0.9	4.3	0.002	2.2	1.3	3.0	<0.001
	3	1.4	−0.1	3.0	0.073	1.4	0.6	2.2	0.001
Peer violence perpetration score	1	2.7	1.5	4.0	<0.001	0.2	−0.3	0.7	0.524
	2	2.9	1.56	4.1	<0.001	0.2	−0.3	0.7	0.420
	3	2.0	0.84	3.2	0.001	−0.1	−0.6	0.4	0.801
School performance‡	1	−0.6	−0.9	−0.2	0.003	−1.2	−1.5	−0.9	<0.001
	2	−0.6	−0.9	−0.2	0.003	−1.2	−1.5	−0.9	<0.001
	3	−0.2	−0.6	0.1	0.207	−1.2	−1.5	−0.9	<0.001
Days of school missed‡	1	0.5	−0.2	1.3	0.157	0.4	−0.03	0.8	0.070
	2	0.5	−0.2	1.2	0.185	0.4	−0.01	0.8	0.058
	3	0.3	−0.4	1.0	0.418	0.4	−0.1	0.8	0.097

**EMD: Estimated mean difference in scores between disabled and not disabled students.** **Model 1**: unadjusted. **Model 2**: adjusted for age and gender. **Model 3**: adjusted for age, gender and other factors. §Dichotomous variable. ‡Model 3 adjusted for hunger score and depression.

In both countries, disability was associated with peer violence victimization, although the significant test was marginal in Pakistan for the fully adjusted model (p = 0.073). In the unadjusted model for Pakistan, peer violence victimization scores for students with a disability were on average 2.6 units higher, and in Afghanistan, they were 2.1 units higher. After adjusting for other factors associated with peer violence victimization, students with a disability have on average higher peer violence victimization scores (Pakistan: EMD = 1.4, p-value = 0.073; Afghanistan: EMD = 1.4, p-value = 0.001).

The relationship between peer violence perpetration and disability was not consistent in the two countries. In Pakistan, disability was associated with significantly higher peer violence perpetration scores (unadjusted EMD = 2.7, p-value<0.001, adjusted EMD = 2.0, p-value<0.001). In Afghanistan, there was no evidence that students with a disability had higher peer violence perpetration scores. In both Pakistan and Afghanistan, school performance was poorer among children with a disability, but this was particularly notable in Afghanistan (adjusted EMD −1.2, p < 0.001). In Pakistan, the difference was not statistically significant in the fully adjusted model (p = 0.207). In neither country was it found that children with disabilities missed more days of school than those without disabilities.

## Discussion

In Afghanistan, the prevalence of disability among the surveyed children was very high and consistent with recent estimates suggesting childhood disability [9]. In Pakistan, it was much lower, and this may have been related to Pakistan, unlike Afghanistan, having special schools for children with disabilities. In both countries, memory/concentration was the most common type of disability, which may be related to the very high prevalence of food insecurity among the students, as well as stress. Previous research in Afghanistan has shown that disability related to mental health conditions was the most highly prevalent [30]. In Afghanistan, difficulty with vision was the second most common type of disability and this may be related to a lack of access to optical services in the study area. Only 2.0% of children have glasses in Afghanistan, compared with 52% of girls and 39% of boys aged 14–17 years in the USA [30,31].

This paper has identified a strong relationship between disability and the greater likelihood of experiencing violence in school settings. In both Pakistan and Afghanistan, disabled students are significantly more likely than their counterparts to experience peer violence victimization. These findings are consistent with the global literature showing that children with disabilities are more vulnerable to verbal abuse, social exclusion and physical violence from their peers than non-disabled children [11,32]. As all as research showing that peer violence victimization is associated with depression, which is a leading cause of disability [33].

Our findings suggest that some of the disability experienced by children is reversible and interventions to prevent children experiencing disability should be given high priority. The high level of memory/concentration-related disability may well largely stem from food insecurity and school meals can make a substantial difference in this respect [34]. Further, some of the disability due to vision may be amenable to eyeglasses in Afghanistan. Our findings also show the important connections between experience of disability and depressive symptoms. The finding that disabled children experienced more depressive symptoms than non-disabled children is consistent with the literature [18,19] and reflects both the vulnerability of disabled students and the disabling effects of depression. Treating depression in children is very challenging when services are limited as they are in Afghanistan, and much of Pakistan, like most other low-income countries.

We have shown that disabled students were more likely to witness family violence than their non-disabled counterparts (e.g. fathers fighting in Pakistan and mothers being beaten by fathers in Afghanistan). This is consistent with the broader literature, suggest that disabled children are more exposed to family violence [12,35]. Some research suggests that exposure to domestic violence can lead to students developing conduct or attention deficits [36,37], and other research highlights the vulnerability of disabled children to mistreatment, neglect, and violence in household settings [11,38].

Consistent with previous findings, disabled students, demonstrated or reported poorer academic performance than non-disabled students [39,40]. This may be directly related to difficulties with memory and concentration. A link between poor school performance and depressive symptoms among disabled students may be intensified in contexts such as Pakistan and Afghanistan, where their pedagogical and school support systems for learning and inclusive education are limited [41,42].

This study has some limitations. As noted in the methodology section, the two studies in Afghanistan and Pakistan the Washington Group on Disability Statistics short set of questions to measure self-reported disability through five domains of functioning. This tool is limited through brevity, which may lead to under-reporting of developmental or psychosocial difficulties. The Washington Group/UNICEF Module on Child Functioning is a more comprehensive measure for disability in children but was finalized too late for inclusion in our questionnaires. Thus, the two study samples may not capture the full range of difficulties among participants. It is not possible to generalize from the study samples due to the nature of their recruitment into intervention evaluation studies. We also recognize that exposure to violence was measured over the past month and this may not capture less frequent or historical violence exposures. The prevalence of disability in Pakistan was 6% and whilst reflecting more than 1 in 20 children had a disability, it is low enough for some associations not to be statistically significant in our research due to small cell size.

## Conclusion

Disability was highly prevalent among children in school in Pakistan and, even more so, in Afghanistan. We have shown that children with disabilities are at greater risk of experiencing and perpetrating violence, witnessing violence at home, and of depression. School-based violence prevention programs are needed to break the cycle of violence and concomitant mental health impacts. These programs need to be sensitive to the increased risk of violence, notably peer violence in schools, faced by children with disabilities. However, the effectiveness of violence prevention interventions in school for children with disabilities, compared to those without, is largely unknown. This is an important avenue for future research.

## Data Availability

Data available at What Works Data repository and can be accessed at: http://medat.samrc.ac.za/index.php/catalog/WW

## Paper context

Previous studies with children have identified the reciprocal relationship between disability and violence but there has not been research on this in contexts with high levels of violence in Central Asia. This study presents two separate, but comparable baseline assessments of disability prevalence and the experience of peer violence in school children in Pakistan and Afghanistan. School settings need to be safe and protective for all children and need to become spaces that help to challenge stigma and harmful social and cultural norms that perpetuate violence towards children with disabilities.
